# Retraction: Protective effect of tanshinone IIA against cardiac hypertrophy in spontaneously hypertensive rats through inhibiting the Cys-C/Wnt signaling pathway

**DOI:** 10.18632/oncotarget.28780

**Published:** 2025-11-14

**Authors:** Jun Feng, Hua-Wen Chen, Li-Juan Pi, Jin Wang, Da-Qian Zhan

**Affiliations:** ^1^Department of Emergency Medicine, Tongji Hospital, Huazhong University of Science and Technology, Wuhan 430030, Hubei, P.R. China

**This article has been retracted:** Oncotarget’s investigation has found that several images were published elsewhere in a different scientific context. Specifically, Figure 2A contains an image also present in Figure 3 of a concurrently published article by a different research group [1], and in Figure 1 of [2]. Both of these articles have been retracted. Furthermore, Figure 2C overlaps with an image from Figure 6B of a later-published, unrelated paper [3], which has also been retracted. The authors have not responded to the journal’s repeated requests to address the concerns raised. As a result, the editors have concluded that the findings presented in this article are invalid, and an editorial decision has been made to retract the paper.

Original article: Oncotarget. 2017; 8:10161–10170. 10161-10170. https://doi.org/10.18632/oncotarget.14328
